# Levosimendan inhibits release of reactive oxygen species in polymorphonuclear leukocytes *in vitro *and in patients with acute heart failure and septic shock: a prospective observational study

**DOI:** 10.1186/cc10307

**Published:** 2011-07-12

**Authors:** Julia Hasslacher, Klaudija Bijuklic, Cristina Bertocchi, Jordan Kountchev, Romuald Bellmann, Stefan Dunzendorfer, Michael Joannidis

**Affiliations:** 1 Intensive Care Unit and Laboratory of Inflammatory Research, Department of Internal Medicine I, Medical University of Innsbruck, Anichstrasse 35, 6020 Innsbruck, Austria

## Abstract

**Introduction:**

Levosimendan is an extensively investigated inodilator showing also cardioprotective and antiinflammatory effects. The aim of our study was to explore the influence of levosimendan on polymorphonuclear leucocytes (PMN), a main source of reactive oxygen species, *in vitro *and in patients with acute heart failure or septic myocardial depression.

**Methods:**

PMN isolated from healthy volunteers were incubated with levosimendan *in vitro*. After stimulation with N-formyl-Met-Leu-Phe (fMLP) or phorbol 12-myristate 13-acetate (PMA) respiratory burst was quantified using a fluorescent dye. Apoptosis and expression of cell adhesion molecules of PMN were measured by flow cytometry. For determination of *in vivo *effects patients with acute heart failure (n = 16) or septic cardiac failure (n = 9) receiving levosimendan treatment were enrolled consecutively. PMN were isolated to measure respiratory burst activity before treatment as well as one and two hours after initiation of levosimendan administration. Furthermore inflammatory, hemodynamic and renal function parameters were obtained.

**Results:**

*In vitro*, levosimendan suppressed respiratory burst activity in fMLP or PMA stimulated PMN in a dose dependent manner by 30 ± 11% (P < 0.001) at 100 ng/mL and by 27 ± 17% (P < 0.001) at 1000 ng/mL respectively. Markers of apoptosis and PMN cell adhesion molecule expression remained unaffected by levosimendan treatment.

*In vivo*, levosimendan treatment for two hours resulted in a significant reduction of PMA stimulated oxidative burst by 45% (P < 0.01) and fMLP stimulated oxidative burst by 49% (P < 0.05) in patients with acute heart failure. In patients suffering from septic shock levosimendan treatment decreased oxidative burst activity in unstimulated, fMLP and PMA stimulated PMN by 48% (P < 0.05), 46% (P < 0.01) and 43% (P < 0.01) respectively.

**Conclusions:**

Levosimendan appears to exert distinct immunomodulatory effects by decreasing oxidative burst activity of PMN. This property might contribute to the previously described cardioprotective effects of the drug.

## Introduction

Recent evidence extended the classic paradigm of acute heart failure as an exclusive problem of low cardiac output to a syndrome comprising exaggerated inflammatory response. This reaction is characterized by complement activation, release of cytokines and production of other inflammatory mediators, which may play a crucial role in the pathogenesis and prognosis of cardiogenic shock [1-3]. Polymorphonuclear leukocytes (PMN) are thought to play a key role in this process by producing myeloperoxidase, which has been shown to be a biomarker of inflammation and oxidative stress as well as an independent predictor of one-year mortality in acute heart failure [4]. Myeloperoxidase is an essential enzyme for the production of reactive oxygen species (ROS), which are involved in many biological processes contributing to the development and progression of heart failure [5]. ROS lead to oxidative damage, cardiomyocyte apoptosis, direct negative inotropic effects and reduced bioavailability of nitric oxide [6,7].

In severe sepsis and septic shock, enhanced neutrophil activation is reflected by higher oxidative burst activity and is associated with increased mortality [8]. Myocardial depression is a frequently recognized manifestation of organ dysfunction in sepsis and can be attributed to several underlying mechanisms, such as endotoxinemia and overwhelming production of cytokines, nitric oxide or ROS, as well as decreased myofibrillar sensitivity to calcium [9-11].

Levosimendan is a Ca^2+ ^sensitizer and inodilator, which has been used successfully in the management of acute heart failure [12]. Additionally, its immunomodulatory and antiapoptotic properties may provide distinct biologic mechanisms that prevent further cytotoxic and hemodynamic consequences of abnormal immune and neurohumoral responses in acute heart failure [13-16]. Experimental data show that levosimendan exerts a protective action by its antioxidant properties and inhibits hydrogen peroxide (H_2_O_2_)-induced apoptotic cell death in cardiomyocytes [17].

Several studies have also addressed the use of levosimendan as a potent inotropic substance in sepsis and septic shock [18-20] showing beneficial effects on systemic hemodynamics and regional perfusion [21] as well as microcirculatory blood flow [22]. A recent study focused on the beneficial combination of levosimendan and glibenclamide in septic shock, where levosimendan was supposed to antagonize cardiodepression and glibenclamide to inhibit sepsis-induced vasodilatory effects [23]. Despite increasing evidence to extend the indication of levosimendan to sepsis-induced myocardial depression in critically ill patients there is still a lack of knowledge about its exact mechanisms of action in this specific clinical setting [24].

The aim of the present study was to investigate antiinflammatory and antioxidative properties of levosimendan by determining its effect in human PMN. For this purpose we examined the *in vitro *effects of levosimendan on the release of ROS, surface expression of adhesion molecules as well as apoptosis in PMN isolated from healthy volunteers. Additionally, we conducted an observational study in critically ill patients treated with levosimendan for acute heart failure or septic shock with sepsis-associated myocardial depression exploring direct drug effects of levosimendan on respiratory burst activity of PMN isolated from these patients.

## Materials and methods

### *In vitro *experiments

#### Preparation of polymorphonuclear leukocytes

PMN were isolated from EDTA treated blood obtained from healthy volunteers. The study protocol was approved by the local Ethics Committee. Written informed consent was obtained from each volunteer. Density gradient centrifugation was performed with Biocoll separating solution (Biochrom AG, Berlin, Germany) followed by hypotonic lysis of contaminating erythrocytes. The cell preparation was resuspended in medium (HBSS (phenol red free, with Ca^2+ ^and Mg^2+^) GIBCO, Invitrogen, Carlsbad, CA, USA) containing 0.05% BSA (Sigma Aldrich, Munich, Germany).

#### Levosimendan incubation

PMN (5 × 10^6^/mL) were incubated at 37°C (5% CO_2 _atmosphere) with medium (i.e. control) or various concentrations of levosimendan (Simdax™, Abbott, Vienna, Austria) ranging from 10 to 1000 ng/mL and then brought to a final volume of 200 μl/well (96-well plate) containing 10 μM 2',7'dichlorodihydrofluoresceindiacetate (H_2_DCFDA; Invitrogen, Carlsbad, CA, USA) in HBSS. Concentrations were chosen from 10 to 1000 ng/mL to cover therapeutical plasma concentrations usually observed after intravenous administration of levosimendan. An infusion rate of 0.1 μg/kg/min was reported to result in a plasma concentration of 35 ± 9 ng/mL at steady state [25]. Time course experiments applying different incubation periods from 30 minutes to 2 hours have revealed a maximum effect already after 30 minutes. Therefore, this incubation period for levosimendan was chosen for all further experiments.

#### Determination of basal and stimulated oxidative burst activity of PMN

PMN were placed in a humified incubator (37°C, 5% CO_2_) for various time intervals with medium (basal activity) or the triggering agents (stimulated activity): 1 μM N-formyl-Met-Leu-Phe (fMLP; Sigma Aldrich, Munich, Germany) or 324 nM phorbol 12-myristate 13-acetate (PMA; Sigma Aldrich, Munich, Germany). Respiratory burst activity was measured using H_2_DCFDA as a fluorochrome. The assay is based on the oxidation of non-fluorescent H_2_DCFDA to highly fluorescent 2'-7' dichlorofluorescein (DCF), both intracellularly and extracellularly. Fluorescent activity was determined at 485 ± 20 nm excitation and 530 ± 25 nm emission wavelengths using a multiwell plate reader (Cytofluor 2350 fluorescence measurement system, Millipore Corp., Billerica, MA, USA).

#### Scavenging of reactive oxygen species

To determine the ability of levosimendan to directly scavenge ROS, hydrogen superoxide (10 μmol/L) was used at room temperature either in medium or in presence of various concentrations of levosimendan (10 to 1000 ng/mL) to create ROS. Generation of ROS was quantified by measuring fluorescence activity of DCF as described above. To exclude effects of autofluorescence experiments were repeated without hydrogen superoxide.

#### Apoptosis of PMN

PMN were preincubated for 30 minutes with levosimendan (10 to 1000 ng/mL) at 37°C in humidified atmosphere and then incubated with medium (i.e. control) or human TNFα (1 ng/mL and 10 ng/mL) for additional four hours. Early apoptosis of PMN was assessed using the human Annexin V-FITC-kit (BenderMedSystems, Vienna, Austria) following the manufacturer's instructions. Fluorescence activated cell sorting (FACS) analysis was performed using a FACSCalibur (BD Biosciences, San Jose, CA, USA). Numbers of apoptotic PMN were calculated as the percentage of gated cells showing positive staining for Annexin V but no uptake of propidium iodide. PMN with uptake of propidium iodide were considered as necrotic cells.

#### Surface expression of CD62L (selectin) and CD18 (integrin) on PMN

PMN (10^6^/mL) were incubated at 37°C with medium (control) or various concentrations of levosimendan (10 to 1000 ng/mL) for 30 minutes. For FACS analyses of cell adhesion molecule expression, freshly prepared PMN were washed twice and stained with monoclonal antibodies (FITC mouse anti-human CD18 or FITC mouse anti-human CD62L) or an isotype control antibody (FITC mouse IgG1k) and kept on ice in the dark for 20 minutes in PBS supplemented with 5% FCS. After additional washing steps, cells were resuspended in PBS and analyzed on a FACSCalibur. All antibodies were purchased from BD Pharmingen (BD Biosciences, San Diego, CA, USA).

### Patient study

#### Patients

Consecutive patients treated with levosimendan were enrolled at the medical ICU of the University Hospital of Innsbruck from January 2008 to January 2010. They were eligible for inclusion if they received levosimendan as intravenous inotropic therapy due to the following criteria:

A) Patients with acute heart failure received levosimendan if they fulfilled at least three of the following six criteria: 1) neurohumeral therapy (diuretics, β-blockers, calcium antagonists, angiotensin-converting enzyme inhibitors, sartans); 2) a history of progredient dyspnea since three days before admission; 3) radiologic signs of pulmonary fluid overload; 4) deterioration of a known chronic heart failure (ejection fraction (EF) <35%); 5) myocardial stunning due to myocardial infarction or mechanical C-reactive protein (CPR); 6) low-output heart failure (EF <35% determined by transthoracic echocardiography).

B) Patients with septic shock (defined according to the criteria of the International Sepsis Definition Conference [26]) suffering from septic cardiac failure were included if they fulfilled the following criteria: 1) at least two of four systemic inflammatory response syndrome (SIRS) criteria; 2) documented or suspected infection; 3) vasopressor support; 4) septic myocardial depression defined as worsening heart failure in the course of sepsis with an EF less than 45% [21].

Preload optimization by fluid administration and/or vasopressor treatment aiming at a mean arterial pressure (MAP) of 65 mmHg or higher were performed in all patients before being considered for levosimendan treatment.

#### Protocol

Levosimendan was infused continuously for 24 hours. Infusion was started at 0.10 μg/kg/min and titrated up to a maximum of 0.15 μg/kg/min, if hemodynamically tolerated, until a cumulative dose of 12.5 mg was reached. Blood samples for oxidative burst measurements were drawn before, one hour and two hours after the start of levosimendan treatment. Preparation of PMN and determination of basal and stimulated respiratory burst activity were performed according to the protocol described above.

Inflammatory markers (leukocyte counts, CRP, procalcitonin, and serum IL-6) as well as renal function parameters (serum creatinine, serum urea, and serum cystatin C) were determined just before the start and at the end of the levosimendan infusion (± four hours). Serum IL-6 levels were measured with an ELISA kit according to the manufacturer's specifications (BD OptiEIA™, Human IL-6 ELISA set, BD Biosciences, San Diego, CA, USA).

Hemodynamic parameters were assessed before and after levosimendan therapy using either transthoracical echocardiography (EF), minimal invasive monitoring (Vigileo, Edwards Life Sciences, Irvine, CA, USA; PICCO_2_, Pulsion Medical Systems AG, Munich, Germany), or a pulmonary artery catheter (Edwards Life Sciences, Irvine, CA, USA). Sequential Organ Failure Assessment score (SOFA) and Acute Physiology And Chronic Health Evaluation (APACHE) II score, catecholamine therapy, continuous renal replacement therapy and ventilatory support were assessed at the day of therapy. ICU survival was recorded for each group. The study protocol was approved by the local Ethics Committee. Written informed consent was obtained from each patient.

### Statistics

*In vitro *data were reported as mean ± standard deviation after verifying normal distribution by the Kolmogorov-Smirnov test. *In vitro *measurements of oxidative burst were analyzed with the repeated - measures analysis of variance followed by Bonferroni post test. *In vivo *data were reported as median and interquartile range. Oxidative burst measurements of patients were compared by the Friedman test followed by Dunn's multiple comparison test. Laboratory and hemodynamic parameters before and after levosimendan treatment were compared with the Mann Whitney U test. Values of *P *less than 0.05 were considered as statistically significant. SPSS software (PASW statistics 18, SPPS Inc., Chicago, Illinois, USA) was used to analyze data.

## Results

### *In vitro *experiments

#### Effect of levosimendan on oxidative burst activity of PMN

**Figure 1:**
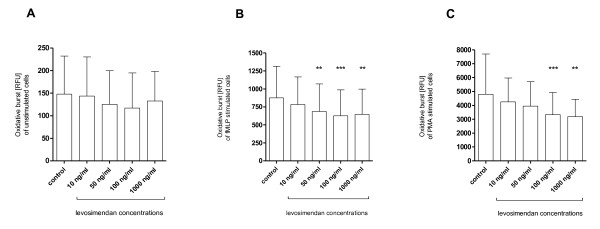
***In vitro *effects of levosimendan on oxidative burst**. Oxidative burst of **(a) **unstimulated, **(b) **N-formyl-Met-Leu-Phe (fMLP) and **(c) **phorbol 12-myristate 13-acetate (PMA) stimulated polymorphonuclear leukocytes (PMN); PMN were incubated with various concentrations of levosimendan ranging from 10 to 1000 ng/mL or control (PMN without levosimendan treatment); Graph shows mean ± standard deviation; n = 11; RFU, relative fluorescence units; ** P < 0.01, *** P < 0.001.

**Figure 2:**
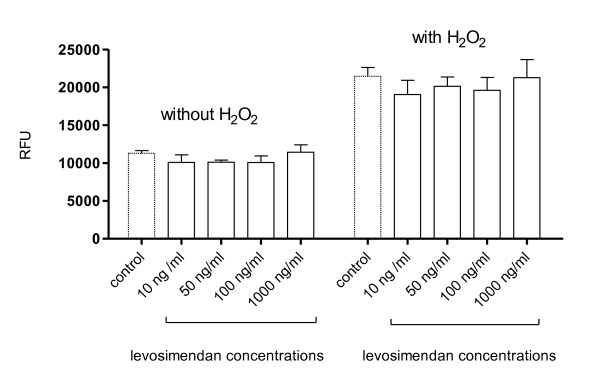
**Scavenging capability of levosimendan**. Oxidation of 2',7'dichlorodihydrofluoresceindiacetate (H_2_DCFDA) to fluorescent 2'-7' dichlorofluorescein (DCF) reflected by fluorescent activity (RFU) was measured in the presence of medium (i.e. control) or various concentrations of levosimendan (10 to 1000 ng/mL) with or without addition of hydrogen peroxide (H_2_O_2_). A scavenging activity of levosimendan would result in a reduction of RFU compared with control. The experiment without hydrogen peroxide was performed to exclude possible autofluorescence of levosimendan. Data are expressed as mean ± standard deviation. RFU, relative fluorescence units.

Incubation with levosimendan resulted in a dose-dependent reduction of oxidative burst reaching statistical significance at 50, 100 and 1000 ng/mL in fMLP-stimulated PMN and at 100 and 1000 ng/mL in PMA-stimulated PMN (Figure 1). A similar trend was found in unstimulated PMN, which, however, was not statistically significant. The maximum effect was seen at 100 ng/mL in fMLP-stimulated PMN showing a reduction of 30 ± 11% (*P *< 0.001) and at 1000 ng/mL in PMA-stimulated PMN showing a reduction of 27 ± 17% (*P *< 0.001). Additional control experiments demonstrated that levosimendan is not able to scavenge free oxygen radicals (Figure 2).

#### Effects of levosimendan on apoptosis of PMN

**Figure 3:**
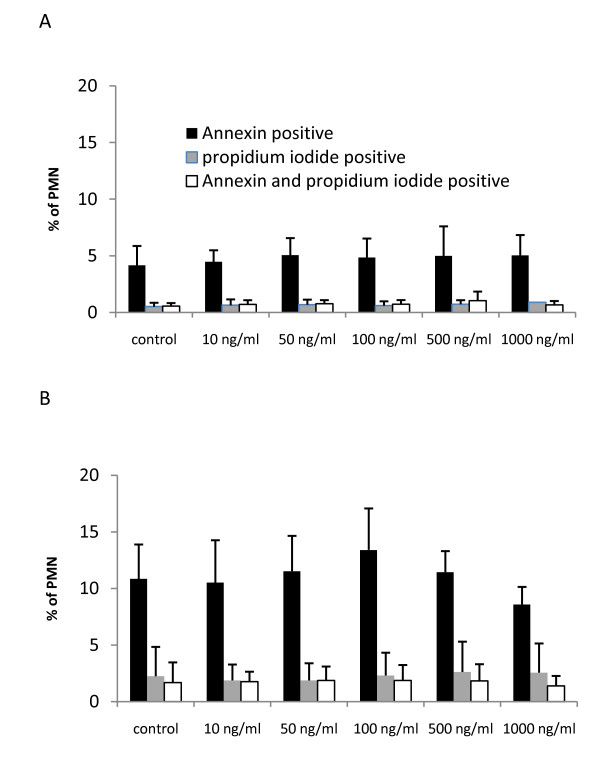
**Apoptosis of PMN incubated with levosimendan**. Graphs show the percentage of annexin positive, propidium iodide positive as well as double positive (annexin and propidium iodide) cells; Data are expressed as mean ± standard deviation; n = 5. **(a) **Polymorphonuclear leukocytes (PMN) were incubated with medium (control) or various concentrations of levosimendan (10 to 1000 ng/mL). **(b) **PMN were preincubated with medium (control) or various concentrations of levosimendan (10 to 1000 ng/mL) and then exposed to TNFα (10 ng/mL).

In healthy volunteers, 4.2 ± 1.7% of untreated PMN were annexin positive (control in Figure 3a). Exposure of PMN to TNFα (10 ng/mL) significantly increased apoptosis to 10.9 ± 3.0% (*P *< 0.01; control in Figure 3b). The rate of necrotic cells did not exceed 1% in untreated and 3% in TNFα-treated cells. Incubation of PMN with levosimendan did not influence the percentage of apototic cells at any concentration (10 to 100 ng/mL; Figure 3a). Preincubation with levosimendan prior to TNFα (10 ng/mL) exposure had no effect on apoptosis induced by TNFα (Figure 3b).

#### Expression of selectins (CD62L) and integrins (CD18) on the surface of PMN

**Figure 4:**
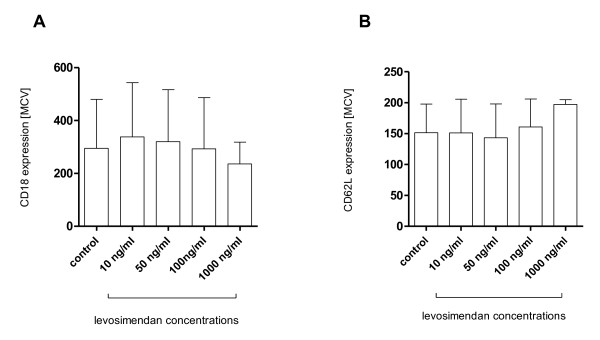
**Expression of cell adhesion molecules**. Surface antigen expression of polymorphonuclear leukocytes (PMN) after incubation with various concentrations of levosimendan (10 to 1000 ng/mL) or medium (i.e. control). **(a) **CD18 (integrin) and **(b) **CD62L (selectin); data are expressed as mean ± standard deviation; n = 4; MCV, mean channel value.

Significant surface expression of integrins (Figure 4a) and selectins (Figure 4b) could be detected in unstimulated PMN. Incubation of PMN with levosimendan (10 ng/mL to 1000 ng/mL) for 30 minutes did not show a significant effect on surface expression of CD62L and CD18.

### Patient study

#### Study population

The study population consisted of 25 consecutive patients including 16 patients with acute heart failure and 9 patients with septic shock showing septic myocardial depression. The demographic data of each group is shown in Table 1, and hemodynamic and laboratory parameters are shown in Tables 2 and 3.

**Table 1:** Patient characteristics

	Acute heart failure (n = 16)	Septic cardiac failure (n = 9)
Mean age	69 (17)	74 (16)
Women	4 (25%)	2 (22%)
SOFA Score	6 (5-10)	13 (11-16)
APACHE II Score	16 (11-19)	20 (15-27)
Patients on catecholamines	7 (44%)	9 (100%)
Patients on mechanical ventilation	7 (44%)	9 (100%)
Patients on CRRT	4 (25%)	8 (89%)
ICU Survival	12 (75%)	2 (22%)

Table shows demographic data of patients with acute heart failure or septic cardiac failure; Age, Sequential Organ Failure Assessment (SOFA) score and Acute Physiology And Chronic Health Evaluation (APACHE) II score are expressed as median (interquartile range); the other parameters are expressed in numbers (%); CRRT, continuous renal replacement therapy.

**Table 2:** Hemodynamic parameters of patients treated with levosimendan

A) Acute heart failure (n = 16)			
**Hemodynamic parameters**	**before levosimendan**	**after levosimendan**	***P *value**

EF (%)	24 (20-29)	38 (29-59)	0.003
MAP (mmHg)	80 (70-90)	75 (70-80)	ns
CVP (mmHg)	8 (6-15)	9 (5-14)	ns
CI (l/min/m^2^)	2.5 (2.3-2.6)	2.7 (2.6-3.3)	0.04
**B) Septic cardiac failure (n = 9)**			
**Hemodynamic parameters**	**before levosimendan**	**after levosimendan**	***P *value**
EF (%)	29 (21-30)	30 (22-38)	ns
MAP (mmHg)	75 (73-80)	75 (75-85)	ns
CVP (mmHg)	9 (5-13)	12 (9-14)	ns
CI (l/min/m^2^)	2.2 (1.9-3.0)	3.2 (2.4-3.9)	ns

Table shows hemodynamic parameters of **(a) **patients with acute heart failure (n = 16) and **(b) **patients with septic cardiac failure (n = 9) before levosimendan treatment and after levosimendan treatment (after 24 hours). Data are expressed as median (interquartile range).

CI, cardiac index; CVP, central venous pressure; EF, ejection fraction; MAP, mean arterial pressure; ns, not significant.

**Table 3:** Laboratory parameters of patients treated with levosimendan

A) Acute heart failure (n = 16)			
**Laboratory parameters**	**before levosimendan**	**after levosimendan**	***P *value**

Leukocytes (G/L)	10.5 (8.3-14.8)	10.5 (7.4-15.7)	ns
CRP (mg/dL)	3.2 (0.6-4.1)	3.1 (1.7-7.4)	ns
Procalcitonin (μg/L)	0.3 (0.1-1.4)	0.4 (0.2-0.8)	ns
Serum IL - 6 (pg/mL)	51 (34-327)	44 (38-136)	ns
Urea (mg/dL)	75 (56-125)	73 (51-150)	ns
Creatinine (mg/dL)	1.6 (0.9-2.4)	1.3 (0.8-2.3)	ns
Serum cystatin C (mg/L)	1.6 (1.3-2.4)	1.7 (1.3-2.6)	ns
**B) Septic cardiac failure (n = 9)**			
**Laboratory parameters**	**before levosimendan**	**after start levosimendan**	***P *value**
Leucocytes (G/L)	10.5 (7.6-16.3)	11.6 (8.2-14.5)	ns
CRP (mg/dL)	14.9 (9.0-21.4)	14.2 (5.3-21.5)	ns
Procalcitonin (μg/L)	4.8 (1.1-26.9)	2.8 (1.1-21.3)	ns
Serum IL-6 (pg/mL)	649 (387-1097)	787 (477-1033)	ns
Urea (mg/dL)	76 (47-128)	82 (46-159)	ns
Creatinine (mg/dL)	1.4 (0.8-2.5)	1.6 (0.7-2.1)	ns
Serum cystatin C (mg/L)	2.3 (2.0-3.1)	2.4 (2.0-3.0)	ns

Table shows laboratory parameters of **(a) **patients with acute heart failure (n = 16) and **(b) **patients with septic cardiac failure (n = 9) before levosimendan treatment and after levosimendan treatment (after 24 hours); Data are expressed as median (interquartile range). CRP, C-reactive protein; ns, not significant.

#### Hemodynamic parameters

Patients with acute heart failure showed a significant increase in EF from 24% (20 to 29%) to 38% (29 to 59%; *P *= 0.003) and cardiac index from 2.5 l/min/m^2 ^(2.3 to 2.6 l/min/m^2^) to 2.7 l/min/m^2 ^(2.6 to 3.3 l/min/m^2^; *P *= 0.04) following levosimendan infusion, whereas hemodynamic parameters in patients with septic shock remained unchanged (Table 2).

#### Vasopressor support

During levosimendan therapy, 7 of 16 patients with acute heart failure required vasopressor support as follows: six received noradrenaline infusion with a dose of 0.26 μg/kg/min (0.12 to 0.40 μg/kg/min) at baseline, 0.24 μg/kg/min (0.08 to 0.36 μg/kg/min) at one hour, and 0.24 μg/kg/min (0.07 to 0.33 μg/kg/min) at two hours of levosimendan treatment. Two patients received dobutamine prior to levosimendan with an average dose of 2.4 μg/kg/min.

In contrast, all patients with septic shock required vasopressor support. Before levosimendan therapy the average dose of noradrenaline administered was 0.27 μg/kg/min (0.23 to 0.39 μg/kg/min), after one hour 0.25 μg/kg/min (0.20 to 0.36 μg/kg/min), and after two hours 0.25 μg/kg/min (0.22 to 0.35 μg/kg/min). Two patients received dobutamine before levosimendan therapy with an average dose of 7.5 μg/kg/min.

#### ICU survival

No differences could be observed in respiratory burst activity, decrease of respiratory burst activity within two hours, or serum IL-6 levels between survivors and non-survivors in patients with acute heart failure or septic shock.

#### Respiratory burst activity of patients treated with levosimendan

##### Patients with acute heart failure (n = 16)

**Figure 5:**
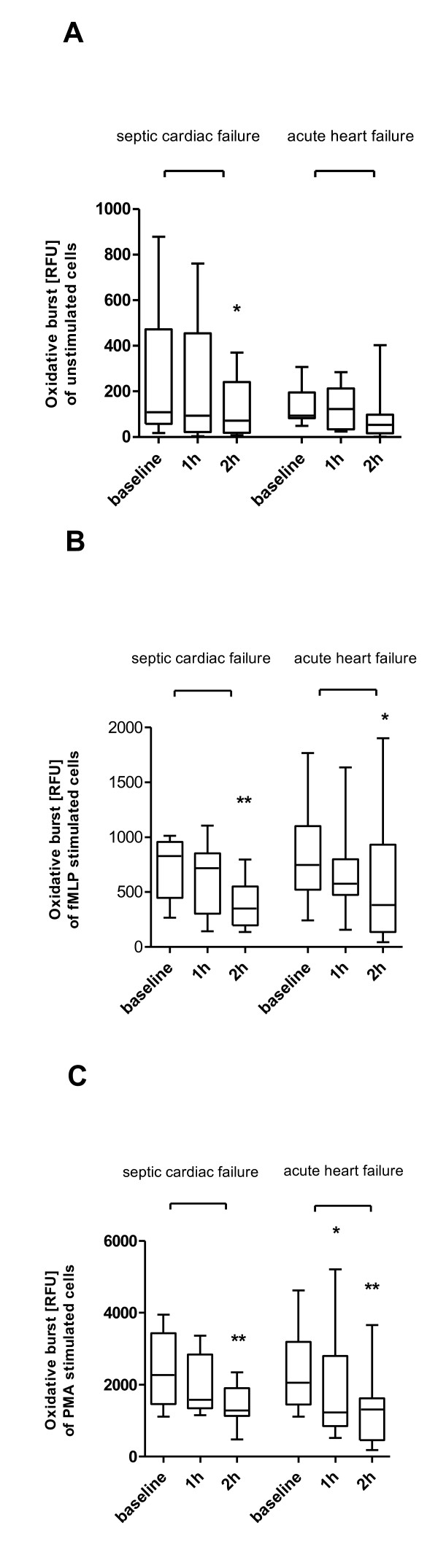
**Oxidative burst activity in patients with acute heart failure or septic shock treated with levosimendan**. Basal oxidative burst and oxidative burst after one hour and two hours of levosimendan infusion in **(a) **unstimulated, **(b) **N-formyl-Met-Leu-Phe (fMLP) and **(c) **phorbol 12-myristate 13-acetate (PMA) stimulated polymorphonuclear leukocytes (PMN) in patients with septic cardiac failure (n = 9) and patients with acute heart failure (n = 16); Graph shows box plots (median and inter quartile range); RFU, relative fluorescence units; * P < 0.05, ** P < 0.01.

There was a significant reduction of oxidative burst from baseline (100%) by 28% (15 to 45%) after one hour (*P *< 0.05) and by 45% (10 to 73%; *P *< 0.01) after two hours of levosimendan infusion in PMA-stimulated PMN and a significant reduction by 49% (-15 to 74%; *P *< 0.05) after two hours in fMLP-stimulated PMN. No significant change was observed in unstimulated cells (Figure 5).

##### Patients with septic shock (n = 9)

In the group of septic patients levosimendan did reduce respiratory burst within two hours by 48% (38 to 65%) in unstimulated PMN (*P *< 0.05), by 46% (32 to 64%) in fMLP-stimulated PMN (*P *< 0.01) and 43% (24 to 53%) in PMA-stimulated PMN (*P *< 0.01; Figure 5).

## Discussion

### Effects of levosimendan on reactive species production of PMN

The present study demonstrates for the first time that levosimendan significantly suppresses ROS production of human PMN isolated from healthy volunteers. This *in vitro *effect could be reproduced in critically ill patients with acute heart failure as well as in patients with septic cardiac failure receiving levosimendan. A significant reduction of respiratory burst was already observed one hour after the start of levosimendan treatment but became even more pronounced after two hours of treatment. In patients with septic shock, oxidative burst activity was reduced in unstimulated as well as in stimulated PMN whereas in patients with acute heart failure only stimulated oxidative burst was suppressed by levosimendan. These findings may indicate a different inflammatory state in the presence of sepsis, where levosimendan does not only reduce stimulated but also basal rates of respiratory burst by about 40 to 50%. Generally higher oxidative burst activities of unstimulated PMN could be observed in critically patients as compared with healthy volunteers.

Based on the time-dependent effect of levosimendan within two hours, we assume a direct mechanism of action responsible for the observed influence on PMN rather than clinical improvement, which usually is not observed within such a short time span. Accordingly, inflammatory markers like leukocyte count, CRP, procalcitonin, and serum IL-6 levels did not change significantly over 24 hours in patients treated with levosimendan. This hypothesis is further substantiated by the results of our *in vitro *experiments where only 30 minutes of levosimendan incubation of PMN isolated from healthy volunteers was sufficient to induce a significant reduction of respiratory burst activity.

Above all, our results support previous findings in patients with advanced heart failure showing that levosimendan does not increase circulating markers of oxidative and nitrosative stress in contrast to placebo [27]. Malondialdehyde, as a marker of oxidative stress, was even significantly reduced by levosimendan compared with dobutamine after five days of treatment [14]. The influence of levosimendan on these markers in patients with septic shock has not been investigated so far. Of notice, the antioxidative and antiinflammtory effects reported cannot be explained by ROS scavenging activity of the drug itself, which we could exclude by our *in vitro *investigation.

### Effects of levosimendan on apoptosis and cell adhesion molecules

Treatment of patients with acute heart failure with levosimendan has been reported to decrease circulating apoptotic markers such as soluble Fas and Fas-ligand [13]. Low concentrations of levosimendan may also prevent cardiac myocytes from apoptotic cell death mediated by ROS after the activation of mitochondrial ATP-sensitive potassium channels [28]. In a mouse model, levosimendan improved ventricular function and inhibited cardiomyocyte apoptosis in severe acute experimental coxsackie virus myocarditis and was discussed as a therapeutic option in patients with viral myocarditis [29]. Therefore, we investigated possible antiapoptotic effects of levosimendan on PMN *in vitro*. In our study, levosimendan had no effect on apoptosis of PMN and could not prevent PMN from TNFα-induced apoptotic cell death.

Activation of PMN is associated with the cell surface expression of selectins and integrins. They play a key role in rolling and adhesion of leukocytes to the endothelium. L-selectin (CD62L), which is highly expressed on resting PMN, is shedded upon cell activation, whereas the expression of integrins (CD18/11b, CR3, or Mac-1 complex) increases rapidly [30-32]. In our study, levosimendan had no influence on adhesion molecule expression of PMN *in vitro*.

### Possible mechanisms of levosimendan responsible for inhibition of respiratory burst activity of PMN

The mechanism by which levosimendan depresses respiratory burst remains speculative. The rapidly detectable effects of levosimendan shortly after the start of infusion into patients with acute heart failure are unlikely the result of hemodynamic improvement. As there was no change in concomitant treatment within this short time span the effect can be attributed to levosimendan alone. As a Ca^2+ ^sensitizer, levosimendan should not lead to substantial changes in intracellular Ca^2+ ^levels. At higher concentrations, however, levosimendan exhibits phosphodiesterase III inhibitory effects, leading to an increase in cyclic adenosine monophosphate, thereby possibly promoting catecholamine effects. Other phosphodiesterase inhibitors such as amrinone and enoximone have already been shown to decrease oxidative burst activity *in vitro *[33]. Furthermore, levosimendan activates Ca^2+^-activated, voltage-sensitive, and ATP-sensitive potassium channels [34-37]. Membrane hyperpolarization in PMN, mediated through potassium channels, may blunt the oxidative burst [38,39]. Thus, reasonable explanations for the inhibitory effect on respiratory burst activity of PMN by levosimendan may be phosphodiesterase III inhibition or decreased ROS production caused by activation of potassium channels.

### Clinical impact

Levosimendan treatment resulted in hemodynamic improvement in patients with acute heart failure [40], which appeared more effective as compared with dobutamine in previous studies [41]. We did also observe a significant increase in EF and cardiac index after 24 hours of levosimendan treatment in patients with acute heart failure. Separate analysis of patients with septic shock showed a similar tendency but did not reach statistical significance. Beneficial effects of levosimendan in septic myocardial depression resulting in an increase in left ventricular EF and cardiac index were reported by Morelli et al. [21].

One explanation for the improvement of cardiac performance may be the reduction of ROS activity from PMN thereby protecting cardiomyocytes from the toxic effects of oxidative stress in addition to its well described mechanism as an inotropic drug [28]. Unfortunately, we could not observe any relation between a decrease in oxidative burst and survival. Further studies are required to demonstrate whether the favorable hemodynamic effects of levosimendan, as also demonstrated in the present study, are clearly potentiated by its anti-inflammatory action.

### Limitations

Our study has several limitations. First of all the patient study was designed as a prospective observational study with a relatively small number of patients but lacking a control group. Therefore, we cannot absolutely exclude that the *in vivo *effects on respiratory burst observed may occur from some other factors than levosimendan. However, by keeping all interventions constant over the two-hour observation period, we tried to minimize this possibility. Secondly, blood concentrations of levosimendan in patients were not measured. However, we used body weight adjusted dosages as recommended by the manufacturer and the early onset of the effect as well as the dose-response relation of respiratory burst inhibition *in vitro *are indicators of a direct drug effect. Although the present study does not allow for firm conclusions to be drawn about the exact mechanism of the inhibition of respiratory burst, scavenging of free oxygen radicals or an apoptotic effect on PMN could be clearly excluded.

## Conclusions

It could be demonstrated for the first time that levosimendan reduces oxidative burst activity of PMN both *in vitro *and in patients with acute heart failure or septic shock with septic myocardial depression. This may contribute to the anticipated cardioprotective effects of the drug.

## Key messages

• Levosimendan significantly reduces oxidative burst activity of PMN from healthy volunteers.

• Immunomodulation (reduced PMN burst activity) by levosimendan can also be observed in critically ill patients suffering from septic shock with septic myocardial depression or acute heart failure.

• Levosimendan as a substance does not exhibit free radical scavenging properties.

## Abbreviations

APACHE II: Acute Physiology And Chronic Health Evaluation II; BSA: bovine serum albumin; CRP: C-reactive protein; DCF: 2'-7' dichlorofluorescein; EF: ejection fraction; ELISA: enzyme-linked immunosorbent assay; FACS: fluorescence activated cell sorting; FCS: fetal calf serum; fMLP: N-formyl-Met-Leu-Phe; H_2_DCFDA: 2',7' dichloro dihydro fluoresceindiacetate; IL: interleukin; MAP: mean arterial pressure; PMA: phorbol 12-myristate 13-acetate; PMN: polymorphonuclear leukocytes; ROS: reactive oxygen species; SOFA score: Sequential Organ Failure Assessment score; TNFα: tumor necrosis factor alpha.

## Competing interests

The authors declare that they have no competing interests.

## Authors' contributions

JH participated in the design of the study protocol, carried out the *in vitro *and *ex vivo *experiments, performed sample and data acquisition, and statistical analysis, and drafted the manuscript. KB and JK conceived the study, performed pilot experiments and helped to draft the manuscript. CB participated in designing the *in vitro *experiments and contributed to the interpretation of the results and drafting of the manuscript. RB and SD participated in patient selection and treatment as well as data and sample acquisition and helped drafting the manuscript. MJ conceived the study, designed the study protocol, supervised and coordinated the study, participated in statistical analysis, data interpretation as well as drafting and finalized the manuscript. All authors read and approved the final manuscript.
